# Comparison of force variables and dynamic strength index between age groups in elite young Brazilian football goalkeepers

**DOI:** 10.3389/fspor.2024.1282214

**Published:** 2024-01-26

**Authors:** Daniel L. Portella, Pedro Jatene, Alex O. Da Silva, Gustavo S. dos Santos, Diogo Monteiro, José E. Teixeira, Luís Branquinho, Ricardo Ferraz, Pedro Forte

**Affiliations:** ^1^ Group of Study and Research in Physical Exercise Science, University of São Caetano do Sul, São Caetano do Sul, Brazil; ^2^ Master Program in Innovation in Higher Education, University of São Caetano do Sul, São Caetano do Sul, Brazil; ^3^ São Paulo F.C., São Paulo, Brazil; ^4^ Cefise Biotecnologia, São Paulo, Brazil; ^5^ Ibrachina F.C., São Paulo, Brazil; ^6^ ESECS—Polytechnic Institute of Leiria, Leiria, Portugal; ^7^ Research Center in Sports, Health and Human Development, Covilhã, Portugal; ^8^ Department of Sports Sciences, Polytechnic Institute of Guarda, Guarda, Portugal; ^9^ Department of Sports Sciences, Polytechnic Institute of Bragança, Bragança, Portugal; ^10^ SPRINT—Sport Physical activity and health Research & Inovation Center, Rio Maior, Portugal; ^11^ CI-ISCE, ISCE Douro, Penafiel, Portugal; ^12^ Agrarian School of Elvas, Polytechnic Institute of Portalegre, Portalegre, Portugal; ^13^ Department of Sports Sciences, University of Beira Interior, Covilhã, Portugal; ^14^ Department of Sports Sciences, Higher Institute of Educational Sciences of the Douro, Penafiel, Portugal; ^15^ LiveWell—Research Centre for Active Living and Wellbeing, Polytechnic Institute of Bragança, Bragança, Portugal

**Keywords:** youth, force, countermovement jump, goalkeeper, soccer

## Abstract

**Introduction:**

The application of muscle force is a determinant of football success as it is inherent to the motor control and sport. The aims of this study are: (1) to describe force variables Isometric Maximal Force (IMF), Concentric Peak Force (CPPF), and Dynamic Strength Index (DSI) in football goalkeepers from different age groups; (2) to compare these variables’ behavior between those groups.

**Methods:**

The sample was formed by 19 youth players (15.97 ± 1.55 years old) from a first-division Brazilian football team. The CPPF and IMF variables were obtained through the Countermovement jump and isometric squat tests, respectively. For data collection, a force plate (Cefise, Brazil) was used with an acquisition frequency of 600 Hz and mono-axial. The DSI was calculated using the ratio between CPPF and IMF. For data analysis, the sample was separated into clusters by age. After the grouping, a descriptive analysis of the data and a comparison between the groups with *p* < 0.05.

**Results:**

The sample was grouped into three groups (GA, GB, and GC) and one of the individuals did not enter the group, totaling 18 individuals in the analyzed sample. The comparison between the ages of the groups showed a significant difference and small and moderate effect size (ES), validating the cluster strategy. The CPPF and IMF variables showed increased values according to chronological age. CPPF showed a significant difference between GA-GB, (ES = very large) GA-GC (ES = very large), and GB-GC (ES = moderate). The IMF variable had significant differences between GA-GB (ES = moderate) and GA-GC (ES = very large). However, DSI showed significant differences only between GA GB (ES = small) and GB-GC (ES = very large).

**Conclusions:**

The CPPF and IMF variables had constant increases and distinct values with an increase according to age, and this did not occur for DSI. The difference between CPPF and IMF compared to DSI bring to light the variability in dynamics and proportionality between muscular force in the concentric phase and maximal force in the isometric regime during the developmental process over chronological age in soccer goalkeepers.

## 1 Introduction

The application of force is a determinant of football success as it is inherent to the motor activity required by the sport (accelerations, decelerations, changes of directions, etc.) (1, 2). In that regard, it is of uttermost value for goalkeepers, who explosively apply force to perform jumps, dives, and saves, hence influencing directly the outcome of matches (3, 4). Thus, the football goalkeeper presents characteristics related to strength, power, agility and power particular in relation to the other field positions. Specifically, quick reactions and agility are crucial for goalkeepers to make rapid movements and save shots effectively (5, 6). Goalkeepers need strength in their arms, legs, and core to generate powerful dives and clears. The ability to catch, parry, or punch the ball safely is a fundamental goalkeeping skill (7). Also, goalkeepers should be adept at stopping shots, whether they come from close range, long-range shots, or set-pieces, requiring an optimal footwork allows goalkeepers to position themselves correctly, move swiftly, and distribute the ball effectively getting the ball back in the right direction (7, 8). Additionally, the goalkeeper's tactical behavior requires a good game reading, decision making and position themselves optimally to cut down angles and be ready for shots in which agility, power and strength are key (7).

The development of explosive force depends on morpho-functional elements such as the increase in the transverse section of skeletal muscle, tendon stiffness, motor unit recruitment (1), and the kinetic and kinematic applied to the action itself (9). Thus, the training tasks should increase goalkeeper skills for fast decisions, direction changes, and movement on the pitch (10). Speed, agility, and response time are all essential characteristics in football, and training methodologies such as speed, agility, and quickness (SAQ) ttraining focuses on building the neuromuscular system to improve these skills (11, 12). The literature has been debating some biomechanical parameters that allow to evaluate concentric, isometric, and dynamic strength parameters because it can be difficult to determine if the trainning stimulus is in the right dose and direction for each age group or maturational (13, 14). Furthermore, explosive power stands out among the conditional and coordinated qualities that make up agility in goalkeeper position (10).

There are appropriate measurements to verify if the application of force is fit for the task. The Dynamic Strength Index (DSI) is a relationship between concentric peak power and isometric maximal strength (IMS) (15–17). The Concentric Phase Peak Force (CPPF) refers to the maximum force exerted by a muscle or group of muscles during the concentric phase of a dynamic movement. The concentric phase is the phase of muscular contraction where the muscle shortens, and it is typically associated with overcoming resistance or lifting a load (15). Additionally, the IMS refers to the maximum force that a muscle or group of muscles can generate during a static, or non-moving, contraction. The young football players often need to maintain stability or hold a specific position against resistance (16). Strength levels tend to increase with age as individuals go through puberty and experience physical development, where playing positions in football may have distinct strength requirements (17). The CPPF is measured through countermovement jumps and presents the force generated at the highest velocity, whereas IMS is measured through isometric half-squats and presents the maximal force generated employing a larger motor unit recruitment (16, 17). The information arising from these tests combined with DSI offers sports coaches and practitioners practical knowledge for elaborating interventions aiming at the improvement of strength-dependent capacities (18, 19).

Force platforms, dynamometers, or other force-measuring devices are commonly used to assess CPPF and IMS. Countermovement jump (CMJ), squat jump (SJ) and isometric squat tests allow for the assessment of players' lower limb strength and power (20, 21). CMJ involves the utilization of the stretch-shortening cycle (SSC), where the pre-stretching of muscles during the countermovement enhances the subsequent force production during the jump. In an SJ, the individual starts from a static squatting position with the knees flexed, and then jumps vertically without any countermovement (22).

However, biological development is a non-linear process that entails different paces of evolution for CPPF and IMS over time, thus affecting DSI itself throughout the chronological age of youth football goalkeepers (1, 23, 24). In this sense, it is of uttermost importance to understand the dynamics of each of these variables to prescribe more accurate hence effective training stimulus (25). Although there is a large amount of research showing distinct correlations between isometric and dynamic strength, less research has been done on how training affects the changes in each kind of strength (18, 19). Previous research showed a wide range in the difference between isometric and dynamic strength improvements. A strong evidence that isometric and dynamic strength represent separate neuromuscular domains (20). Specifically, the goalkeepers may have different strength demands compared to outfield players, however little is known about the normality values for DSI indexes in elite young Brazilian football goalkeepers. Thus, the aims of this study were: (1) to describe force variables IMS, CPPF and DSI in football goalkeepers from different age groups; (2) to compare these variables’ behavior between those groups.

## 2 Methods

### 2.1 Sample

This study had a cross-sectional observational prospective design with descriptive and comparative purposes. The sample was formed by 19 academy players (age = 15.97 ± 1.55 years; height = 184.28 m ± 6.22; body mass = 74.94 ± 7.38 kg/m^2^) from an elite Brazilian football club competing with its professional team in the highest national division as well as in continental tournaments. The study was conducted in accordance with the Declaration of Helsinki ethics guidelines and protocols from the local Ethical Committee of University of São Caetano do Sul, Brazil.

### 2.2 Inclusion criteria

The sample was selected according to the following inclusion criteria: (i) athletes had to be goalkeepers between 13 and 20 years old; (ii) duly affiliated to their club through registration in the Brazilian Confederation of Football (CBF); (iii) young football players performed all data collection tests. Non-compliance with either inclusion criterion represented the subject's removal from the convenience sample.

### 2.3 Data collection

Data were collected between 8 and 11 AM (GMT -3 h) on two subsequent days. Football players from U17 and U20 were tested on the first day, whereas athletes from U14, U16, and U17 were tested the day after. All of them undertook a 48 h period of rest before the test. For data collection, for both the IMS variable and CPPF variable, a force plate (Cefise, Brazil) was used with an acquisition frequency of 600 Hz and monoaxial. In order to calculate body weight for the countermovement jump (CMJ) (vertical force averaged over one second), individuals were required to remain stationary for the first second of data collection (15, 16). With a minute of rest in between each trial, each subject completed three CMJ trials using their maximum effort. With their hands on their hips, the subjects were encouraged to execute the leaps as quickly and high as they could (15, 19). CPPF, IMS and DSI have been previously validated in youth football insights as critical force variables (18, 26).

### 2.4 Statistical procedures

Descriptive statistics, the Kolmogorov–Smirnov and Levene's test were used to assess the normality and homogeneity. Data are presented as the mean ± one standard deviation (SD). A cluster modeling was performed using single linkage for rescaled distance cluster combination, represented by a dendogram. Based on the similarities between a set of points or subjects, the k-means defines a centroid, or the mean of the cluster. To guarantee a logical comparison of data sets with various magnitudes and/or units, standardized *z*-scores were employed. To determine how many clusters should be kept for analysis, the elbow approach was applied (27). Additionally, a one-way analysis of variance (ANOVA) for repeated measures were tested to identify differences between age group with turkey's *post-hoc* tests for localized effects. The effect size index (eta square: *η*^2^) was computed and interpreted as: (i) without effect if 0 < *η*^2^ ≤ 0.04; (ii) minimum if 0.04 < *η*^2^ ≤ 0.25; (iii) moderate if 0.25 < *η*^2^ ≤ 0.64; and (iv) strong if *η*^2^ > 0.64. For pairwise comparison, using the Tukey *post hocs*, the standardized effect sizes (ES) were calculated with Cohen's d, classified as: without effect if *d* < 0.2, moderate effect if 0.2 > *d* ≥ 0.5 and strong effect if *d* > 0.5. Statistical significance was set at *p* < 0.05 (28). All statistical analyses were conducted using SPSS for Windows Version 22.0 (SPSS Inc., Chicago, IL, USA) and Microsoft Excel® spreadsheet (Microsoft Corporation, U.S.).

## 3 Results

**Figure 1:**
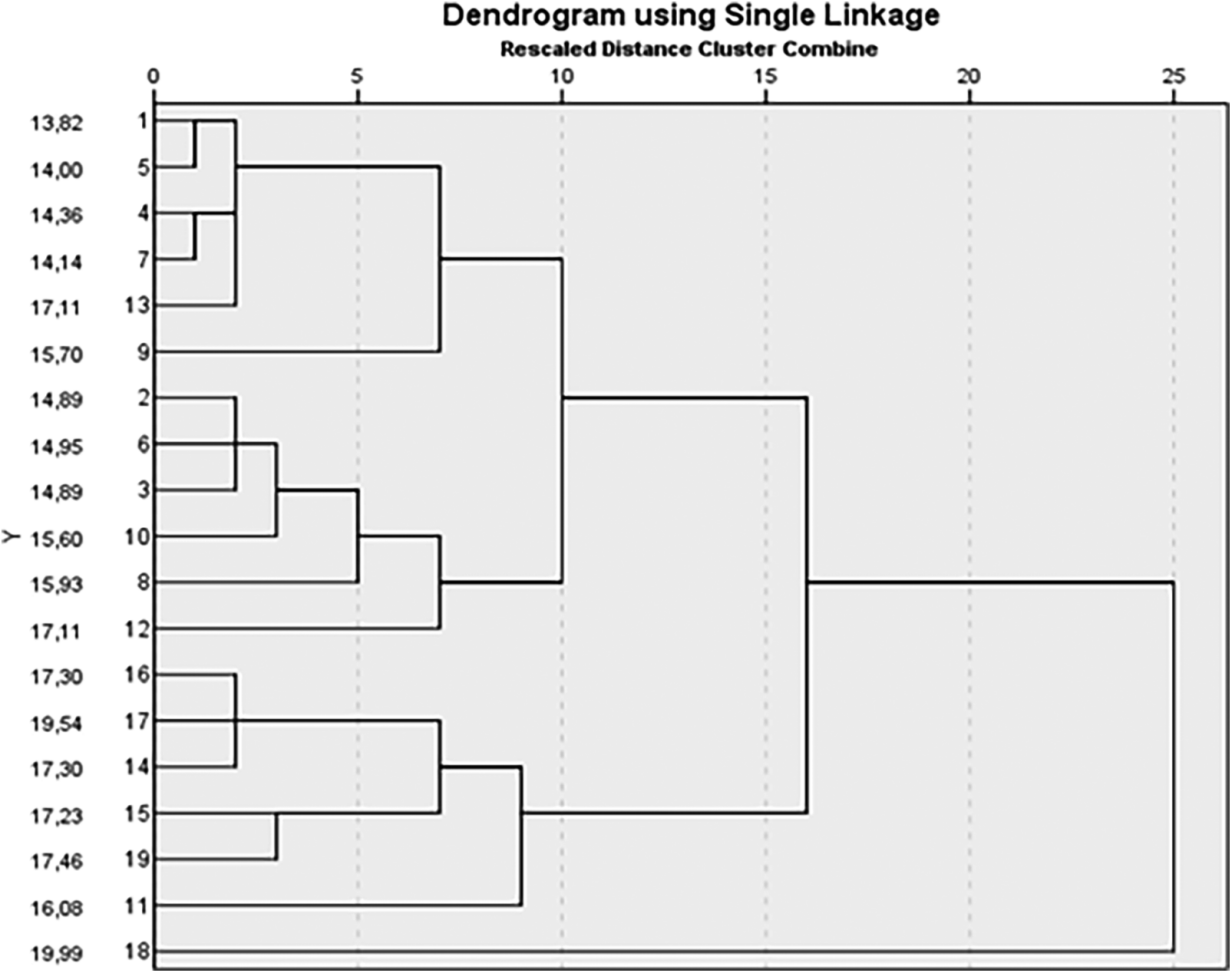
Dendrogram using Single Linkage for rescaled distance cluster combination.

Figure 1 presented the distance cluster combination using a Single Linkage Dendrogram. The DSI of cluster A and B athletes (younger) occurs at the expense of a concentric peak force proportionally greater than the isometric peak force. Unlike the younger clusters (A and B), cluster C presents its DSI values because of a higher IMS proportional to the CPPF.

Table 1 showed the descriptive statistic for age, body mass, height, CPPF, IMS, and DSI from Clusters A, B, and C.

**Table 1:** Age, body mass, height, concentric phase peak force, isometric maximal strength, and dynamic strength index from under 14 to under 20 soccer players.

Clusters		Age (years)	Body mass (Kg)	Height (cm)	Concentric phase peak force (*N*)	Isometric maximal strength (*N*)	DSI
Cluster A (*n* = 6)	Mean	14.86	66.53	178.67	1,431.60	1,845.24	0.78
	SD	1.29	3.94	6.68	63.36	149.25	0.09
	Min	13.82	61.00	166.0	1,371.64	1,715.29	0.65
	Max	17.11	71.20	186.0	1,550.84	2,114.41	0.90
Cluster B (*n* = 6)	Mean	15.56	77.80	187.83	1,773.98	2,111.45	0.85
	SD	0.88	3.78	3.55	98.61	203.45	0.11
	Min	14.89	72.50	183.0	1,610.08	1,863.83	0.68
	Max	17.11	88.20	192.0	1,872.37	2,369.51	0.98
Cluster C (*n* = 6)	Mean	17.48	80.50	186.33	1,965.65	2,972.61	0.66
	SD	1.13	4.87	4.03	142.02	231.15	0.04
	Min	16.09	75.00	180.0	1,830.78	2,692.20	0.60
	Max	19.54	88.20	192.0	2,174.34	3,336.60	0.70
Total (*n *= 18)	Mean	15.97	74.94	184.28	1,723.74	2,309.77	0.76
	SD	1.55	7.38	6.22	248.27	528.73	0.11
	Min	13.82	61.00	166.0	1,371.64	1,715.29	0.60
	Max	19.54	88.20	193.0	2,174.34	3,336.60	0.98

DSI, dynamic strength index; Max, maximum; SD, standard deviation.

There are differences between the performance variables by age clusters (*F* = 5.92–53.38, *p *< 0.001 to *p *< 0.05*, η^2 ^= *0.72 to 0.96*)* with significant pairwise on all variables. Pairwise comparison confirmed significant differences between three age groups with small to very large effect size (ES) (*d = *0.63–5.79) (Table 2).

**Table 2:** Significant peak force, isometric maximal strength, and dynamic strength index from under 14 to under 20 soccer players.

Variable	ANOVA				Pairwise comparison	
	M ± SD	F	*p*	*η* ^2^	*post hoc*	d Cohen
Age (years)	15.97 ± 1.55	8.99	0.003	0.96	A ↔ C* B ↔ C**	0.63 1.90
Body mass (Kg)	74.94 ± 7.38	5.92	0.013	0.90	A ↔ B* A ↔ C*	2.91 3.15
Height (cm)	184.28 ± 6.22	18.48	<0.001	0.72	A ↔ B* A ↔ C**	1.71 1.39
Concentric phase peak force (*N*)	1,723.74 ± 248.27	38.86	<0.001	0.91	A ↔ B* A ↔ C* B ↔ C**	4.13 4.86 1.57
Isometric maximal strength (*N*)	2,309.77 ± 528.73	53.38	<0.001	0.89	A ↔ B* A ↔ C*	1.49 5.79
DSI	0.76 ± 0.11	7.42	0.006	0.91	A ↔ B* B ↔ C**	0.70 2.30

Statistical differences were verified as: *p < 0.001; **p < 0.05.

DSI, dynamic strength index; M, mean; SD, standard deviation.

**Figure 2:**
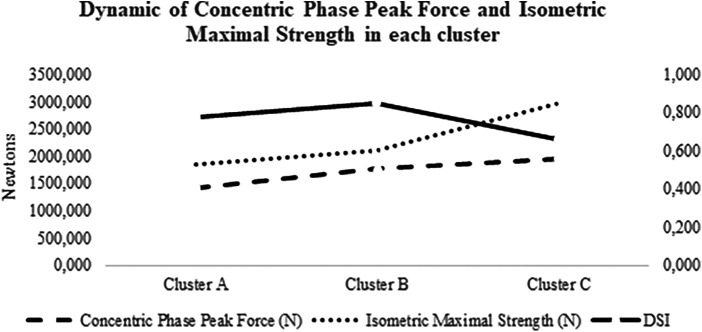
Dynamic evolution of concentric phase peak force and isometric maximal strength in each cluster group.

The evolution dynamics of the CPPF and IMS occur increasingly in the course of age, unlike the variable DSI (Figure 2).

## 4 Discussion

This study is the first to address DSI in a population of young athletes, that is, under 19 years of age, in the present study, in particular for goalkeepers. This is of great importance, as these are variables with precise acquisition as well as tools such as protocols. The main results of the study were: (1) The concentric action was statistically different between all groups; (2) peak isometric strength did not show statistical difference between groups B and C; (3) DSI appears as a non-linear variable between groups according to age group.

The force peak in the concentric action showed an incremental dynamic from groups A to B and from groups B to C, with moderate effect sizes or above. This demonstrates that over chronological age this variable behaves linearly and positively concerning the increase in the values obtained. This is largely explained by two points, the first being the natural increase in body mass and in particular muscle mass that occurs during adolescence as a result of biological maturation (15, 29, 30). The second point is the improvement of muscle function as force production through concentric action, both due to the training stimuli (31, 32) and the biological development arising from the increase in the hormone secretion rate (33–35).

The IMS showed a dynamic similar to the CPPF, however, the differences demonstrated were significant only between groups A and B as well as A and C, and the effect size between A and C was very large and between A and B moderate. A possible explanation for why the same dynamics did not occur concerning the significant differences in the IMS variable compared to the CPPF variable is the ability to recruit muscle fibers and muscle tissue maturity. The application of maximum force, whether in an isometric form or any other regime of muscular contraction, demonstrates a very high dependence on the recruitment of muscular fibers (1, 36, 37). Thus, skeletal muscle tissue maturity occurs chronologically at a later stage and may not significantly interfere with this variable in which the recruitment of muscle fibers is a priority (1, 32).

On the other hand, the DSI variable showed significant differences between groups A and B as well as between B and C, with the effect size between A and B being small and between B and C being very large. However, between the extreme groups A and C there was no such difference. It is known that DSI occurs due to a relationship between two variables that do not have the same morphofunctional dependencies. The CPPF occurs a lot because of the ability to produce force in the concentric action, which implies a morphological issue (18). On the other hand, IMS is primarily influenced by neural issues. Thus, fiber recruitment was stable between groups B and C as opposed to force production through concentric action (16).

Several collinear and multi-independent factors such as training history, skill level and the specific demands of the position can contribute to the variability of strength variables (DSI, CPPF and IMS) (38). Football player with a longer training experience within a regular and well-structured training programs tend to have better strength levels over time (16). Also, higher technical proficiency may be able to better translate their strength into sport-specific movements. Skilled young football often display more efficient neuromuscular coordination (39). Moreover, different playing positions in football have distinct physical demands (16). For example, defenders may benefit from isometric strength for physical challenges and stability, while forwards may require explosive strength for sprints and shots (16). However, the positional effect can be overlooked when reporting on young football players (40). However, the specificity of the goalkeeper position tends to differ from the other positional roles (8). This may partly explain the fact that CPPF and IMF variables had constant increases and distinct values with an increase according to age, whereas the DSI has not increased.

In the adult population, these variables can develop linearly and in parallel, however these adaptations of different natures (neural and morphological) occur asynchronously, that is, at different times in the young population (15, 19). Therefore, the DSI that is measured by a relationship between the two variables may show non-linear results of improvements. This observation resulting from the results presented points to the longitudinal follow-up of these variables to understand the cause-and-effect relationship in the performance of young athletes (41). In addition, the DSI is a variable that can be used as a reference for understanding training content needs after collections and over the years (18).

The practical applications of this study are as follows (8, 3): (1) stability and positioning: Isometric strength is crucial for goalkeepers to maintain stability and hold positions, especially when preparing for a shot or making a save. Developing IMF can contribute to improved body control and balance; (2) shot power: concentric strength is essential for explosive movements, such as diving to reach a ball or generating power in a throw or kick. Goalkeepers with higher CPPF may exhibit greater shot-stopping ability and efficiency in dynamic actions; (3) performance analysis: DSI, if used as a measure of dynamic force production, could be valuable for analyzing the goalkeeper's ability to generate force in real-game situations. This can aid in identifying strengths and areas for improvement in dynamic movements; (4) Integrated Training Programs that incorporates isometric and concentric strength training along with dynamic movements is beneficial. This can include exercises focusing on stability, power, agility, and reaction time; (5) Injury Prevention: understanding force variables can help identify potential muscular imbalances, allowing for targeted training to prevent injuries. Goalkeepers often engage in asymmetrical movements, and a balanced strength program can mitigate the risk of overuse injuries; (6) individualized Programs: Tailoring training programs based on assessments of IMF, CPPF, and DSI can help in creating individualized strength and conditioning plans. Periodic assessments can track progress and guide adjustments to the training regimen.

The present study brings the first analysis in the literature of the behavior of these variables in the young athlete population. This research has a prospective cross-sectional observational study design, which allows more hypotheses to be generated than tested. Furthermore, some methodological limitations should be considered when reading this article. Current training data reflect only Brazilian football goalkeepers and hence cannot be extended to other contexts. Hence, more analyses are required for this purpose, with a wider follow-up and comparing different competition level and age groups. In addition, it points to the importance of understanding the cause variables and not just looking at the outcome variables. Understanding the behavior for later use is an extremely positive point. For future studies, there is a need to include biological maturation as an adjustment variable to increasingly understand the behavior of force applications in young Brazilian football players. Also, the influence of plyometric training, SAQ or SSG strategies on the development of strength variables in young Brazilian football players should be studied from an intervention and clinical trial perspective, controlling for other variables such as the season phase (39), maturational variables (25) and the player's starting status (41).

## 5 Conclusions

The CPPF and IMF variables had constant increases and distinct values with an increase according to age, and this did not occur for DSI. The differences for DSI compared to CPPF and IMF bring to light the variability in dynamics and proportionality between muscular force in the concentric phase and maximal force in the isometric regime during the developmental process over chronological age in soccer goalkeepers.

## Data Availability

The raw data supporting the conclusions of this article will be made available by the authors, without undue reservation.
